# THE HEALTH IMPACT OF SCAMS

**DOI:** 10.1093/geroni/igz038.2781

**Published:** 2019-11-08

**Authors:** Jan Bailey, Paul Kingston, Louise Taylor, Louise Eost-Telling

**Affiliations:** University of Chester, Chester, England, United Kingdom

## Abstract

This presentation will offer new and alternate insights into ‘scams’ and the health effects of fraud on older people. It reports data captured from a Mass Observation Project “Directive” focusing on scams and their impact on individuals. Eighty “Observers’” aged 50 and over responded to the “Directive”. Responses indicate that falling victim to a scam may have negative impacts on individuals’ mental wellbeing, self-esteem and relationships with others. Data analysis also identified that fear of victimisation can also affect individuals, resulting in worry, anxiety and maladaptive coping strategies. Offering a sociology of health perspective, we will focus is on these health impacts of scams and the legitimisation of the issue as a socio-political problem. We will also highlight additional important areas for consideration, such as the absence of a common understanding of the concept and nomenclature of ‘scam’, and the ‘vagaries of scams’ by presenting a typology of scams.

